# Predictability of Urinary CD80 in the Relapse of Primary Nephrotic Syndrome

**DOI:** 10.1155/2017/9429314

**Published:** 2017-08-29

**Authors:** Juan Liao, Xiao-Chuan Wu, Qia Cheng, Can-Lin Li, Zhu-Wen Yi, Yan Cao, Lan-Jun Shuai

**Affiliations:** Department of Pediatric Nephrology, Children's Medical Center, The Second Xiangya Hospital, Central South University, No. 139 Middle Renmin Road, Changsha, Hunan 410011, China

## Abstract

**Purpose:**

The current study is aimed at investigating whether urinary CD80 is reliable to predict the recurrence of pediatric PNS.

**Materials and Methods:**

A total of 128 children, 105 males and 23 females, were enrolled in this study. Urinary samples were collected from SSNS and SRNS patients and 25 healthy children as controls. Urinary CD80 was measured by ELISA and adjusted for urinary creatinine excretion.

**Results:**

Urinary CD80 in relapse stage of SSNS was significantly higher, and the urinary CD80 of paired relapse and remission stages of each SSNS patient were also significantly different. No significant difference was found between the urinary CD80 in SRNS relapse group, SRNS remission group, and the control group. Similarly, there was no significant difference between frequent SSNS and not frequent SSNS in remission group, as well as the relapse group. There is no correlation between urinary CD80 and 24-hour urinary protein.

**Conclusion:**

The increase of urinary CD80 was closely associated with the relapse of SSNS but was not related to the frequency of relapse. The urinary CD80 changes of concentration were reliable to predict the recurrence of SSNS. However, it cannot be used to predicate the frequent recurrence of PNS.

## 1. Introduction

Primary nephrotic syndrome (PNS) is the most common glomerular disease among 2–6-year-old preschool children. Glucocorticoids remain to be the major drugs for its treatment, which can alleviate PNS in 90% of patients. However, approximately 76%–93% will show recurrence, while 57% of the recurrence will be frequent or glucocorticoid-dependent [1, 2]. Therefore, suppression of PNS recurrence is difficult, and hence it is important to find the predictable factors associated with PNS recurrence, which holds great importance.

The mechanism of PNS pathogenesis remains to be clarified. In 1974, Shalhoub [3] in his study proposed that the minimal change disease (MCD) that causes nephrotic syndrome is related to the malfunction of T-cells. Molecular investigations demonstrated that the ratio of Th1 and Th2 and the ratio of Treg and Th17 were imbalanced. The cells secreting proinflammatory cytokines such as Th2 and Th17 were highly expressed, while cells secreting anti-inhibitory cytokines such as Th1 and Treg showed lower expressions [4–8]. However, the underlying mechanism of T-cell malfunction in PNS remains to be unclear, and it is speculated to be associated with adaptive immunology. Clinically, infection of upper respiratory tract is the leading cause of PNS, which activates the innate immunity. To date, several studies have demonstrated that lipopolysaccharide (LPS) can be combined with Toll-like receptors (TLRs) and induce the expression of CD80 on the surface of podocytes, which are related to the development of PNS [9, 10].

As a member of the immunoglobulins superfamily, CD80 which is also known as B7-1 with a molecular weight of 53 kDa plays an important role as a synergetic costimulatory factor of antigen presenting cells (APCs). In a monomer form, it is highly expressed in the activated B-cells, T-cells, macrophages, peripheral mononuclear cells, and dendritic cells [11]. The synergetic costimulatory effect of CD80 is very important in activating primitive T-cells, and it is a prerequisite in the activation of adaptive immune responses [12, 13]. In 2004, Reiser et al. [9] found that podocytes can express CD80* in vitro* in drug induction, immunologic injury, and bacterial toxins, and it can induce urine protein. At the same time, animal experiment demonstrated that the expression of CD80 was significantly elevated in the podocytes of rats when they were exposed to low-dose LPS [9]. Moreover, higher expression of CD80 can lead to the reorganization of actin in podocytes, breakdown of podocytes' structures, and therefore elevation of the podocyte permeability of filtration membranes resulting in proteinuria [14]. On the contrary, knockout of CD80 can help in the avoidance of LPS injury [9]. This indicated that higher expression of CD80 in podocytes induced by LPS is associated with proteinuria. Moreover, several clinical investigations found that the excretion of CD80 in the recurrent phase of MCD is higher than that of normal children, but the excretion of CD80 in MCD remission phase, recurrent phase, and remission phase of focal segmental glomerulosclerosis (FSGS) was not significantly different compared to the control groups [15–18].

Most of the MCD patients are sensitive to glucocorticoids, and 58% of steroid-sensitive nephrotic syndrome (SSNS) patients will incur recurrence of PNS [19]. To date, the expression of CD80 is significantly elevated during the recurrent phase of MCD, but it is normal during the remission phase of MCD and in FSGS [17]. In our study, it is speculated that CD80 is associated with the recurrence of SSNS, and its expression could be elevated in the recurrent phase of SSNS but returns to the normal during the remission phase. Further, the concentration of urinary CD80 is not related to steroid-resistant nephrotic syndrome (SRNS). Therefore, through comparing CD80 of urine in recurrent and remission phases of SSNS and SRNS, the present study aimed to investigate the potential relationship between CD80 expression in the urine and the recurrence of PNS and investigate whether urinary CD80 can be the predictor of PNS recurrence.

## 2. Methods

### 2.1. Patients

A total of 128 children, 105 males and 23 females, were enrolled in this study. There were 49 SSNS patients in relapse stage, 64 SSNS patients in remission stage, and 15 SRNS patients. Of all the SSNS patients in the relapse stage, 31 were frequent relapse and 18 were nonfrequent relapse patients. Of all the SSNS patients in the remission stage, 26 were frequent relapse and 38 were nonfrequent relapse patients. Of all the SRNS patients, 7 were in relapse and 8 were in remission stages. Moreover, 25 normal children undergoing physical examination were taken as the control group.

### 2.2. Definitions

Patients were considered in relapse if they had urinary protein score of 3+ or 4+ for three consecutive days, 24-hour urinary protein ≥ 50 (mg/kg), and urinary protein/creatinine (mg/mg) ratio of ≥2.0. Remission was defined as patients having normal blood biochemistry and urinary protein. SSNS refers to the patients who are treated by enough prednisone for four weeks, whose urine protein turns to be negative, while SRNS is opposite to SSNS. Frequent relapse means recurrence is more than three times in a year or two times in half a year.

### 2.3. Urine Samples

A 10 mL test sample was collected from the midstream fresh urine of each child in the morning and then was centrifuged for not more than an hour after collection at 1,500 ×g for 15 min at 4°C to remove cell debris and particulate matter. Next, all the samples were delivered to sterilized EP tubes and preserved in a −80°C refrigerator and brought to room temperature before use. All samples were stored for a minimum of one day and a maximum of two months before test.

### 2.4. Urinary CD80 Measurements

Urinary CD80 was measured using a commercially available enzyme-linked immunosorbent assay (ELISA) kit (USCN Life Science, Wuhan, China), which was adjusted for urinary creatinine excretion. Urinary protein, creatinine, and serum albumin were measured using an autoanalyzer.

### 2.5. Statistical Analyses

Data graphics and statistical analysis were performed with GraphPad Prism 5 and SPSS (version 23.0 for Windows; SPSS, Chicago, IL, USA).

Data measurement was expressed as mean ± standard deviation (*X* ± *S*) and Kolmogorov-Smirnov test was used to test the normal distribution, and homogeneity of variance test was used to test homogeneity of variances. For the normally distributed variances, *t*-test or one-way ANOVA was used for comparison. For the variances that were not normally distributed, Mann–Whitney test or Kruskal-Wallis *H* test was used. Wilcoxon test was used for comparison between paired groups. The comparison between groups of data enumeration was conducted by *C*2 test, and the relationship between variances was analyzed by Pearson's correlation analysis. In all the statistical analyses, *P* value <0.05 was considered to be statistically significant.

## 3. Results

Statistical analyses of general clinical data of each group were collected and are shown in Tables 1–3.

### 3.1. Urinary CD80 Excretion in SSNS Patients in Relapse and in Remission and Control Groups (Figure 1)

The concentration of urinary CD80 in relapse stage of SSNS (511.30 ± 36.17 ng/g creatinine) was significantly higher than that in remission stage of SSNS (56.52 ± 34.43 ng/g creatinine) (*P* < 0.001) and the control (55.77 ± 19.23 ng/g creatinine) groups (*P* < 0.001). On the contrary, there was no significance for the comparison between the concentrations of CD80 in remission stage of SSNS and the control group (*P* > 0.05).

### 3.2. Urinary CD80 Excretion in the Same Patients in Relapse and in Remission Stages (Figure 2)

Comparison of urinary CD80 of paired relapse and remission stages of each SSNS patient showed statistical significance. The concentrations of urinary CD80 are 496.51 ± 260.80 ng/g creatinine and 44.55 ± 19.81 ng/g creatinine, respectively (*P* = 0.02).

### 3.3. Urinary CD80 Excretion in SRNS Patients in Relapse and in Remission and Control Groups (Figure 3)

No significant difference of urinary CD80 was observed between and among SSNS patients in relapse and in remission and controls: 76.89 ± 64.94 ng/g creatinine, 80.71 ± 93.84 ng/g creatinine, and 55.77 ± 19.23 ng/creatinine, respectively (*P* = 0.96).

### 3.4. Urinary CD80 Excretion in Relapse Group and That in Remission between the Frequent SSNS and Not Frequent SSNS Groups (Figure 4)

Comparison of urinary CD80 between frequent SSNS in relapse group (509.63 ± 278.44 ng/g creatinine) and that in remission group (51.23 ± 37.31 ng/g creatinine) was significant (*P* < 0.001). Comparison of urinary CD80 between frequent SSNS in relapse group and not frequent SSNS in relapse group (514.13 ± 210.24 ng/g creatinine) showed no significance (*P* = 0.70). Similarly, comparison of urinary CD80 between not frequent SSNS in relapse group and that in remission group (62.62 ± 34.29 ng/g creatinine) was significant (*P* < 0.01). Such comparison between frequent SSNS in remission group (51.23 ± 37.31 ng/g creatinine) and not frequent SSNS in remission group showed no significance (*P* = 0.14).

### 3.5. Urinary CD80 and 24-Hour Urinary Protein (Figure 5)

There was no correlation observed between urinary CD80 and 24-hour urinary protein (*Z* = −2.366, *P* > 0.05).

## 4. Discussion

The prognosis of PNS is generally related to the susceptibility of glucocorticoids and the frequency of recurrence. According to the various effects of treatment, PNS can be classified into SSNS, SRNS, and steroid-dependent nephrotic syndrome (SDNS). It was reported that 58% of SSNS could recur if the medication was reduced or ceased [19]. Pertaining to SRNS and SDNS, additional administration of immunosuppressors was needed. Although the application of immunosuppressors is effective, the high risk of recurrent PNS induced by the infection or cessation or reduction of medication is still intractable.

Previous literature found that the concentration of urine CD80 was significantly elevated in the recurrent phase of MCD but remained to be normal in the recurrent and remission phases of other types of PNS [9]. MCD and FSGS are the most common PNS types, and therefore it is speculated that CD80 can be used for identifying MCD and FSGS instead of renal biopsy [17, 18]. Therefore, the current study was aimed at investigating whether the changes of urinary CD80 concentration were reliable to predict the recurrence of PNS.

CD80 is expressed on the surface of APCs, which occupied an important position and played an important role in the activation of naive T-cells. Recent studies indicated that podocytes could perform similarly to APCs in the pathological changes, which made them vulnerable to the modified reaction of the immune system, and expression of CD80 is also related to MCD [20, 21]. As an important costimulatory factor of antigen presenting cells, CD80 can be combined with CD28 and provides the second signal to activate T-cells, which in turn plays an important role in initiating adaptive immune response.

Garin et al. [15, 17] found that urine CD80 could be elevated in the recurrent phase of MCD but remained normal in the remission phase of MCD and other types of PNS including FSGS and mesangial proliferative glomerulonephritis (MsPGN). It is speculated that CD80 is derived from podocytes because (1) in the recurrent and remission phase of MCD the blood CD80 is normal, and therefore the urine CD80 does not come from the APCs into blood; (2) immunofluorescence assay verified that CD80 was expressed by podocytes; (3) the molecular weight of CD80 is 53 kDa, which is the same as that of CD80 on the membrane, rather than the soluble CD80 which is of 23 kDa [17, 22].

Shimada et al. proposed “two hits” theory to describe the mechanism of MCD. Accordingly, the first hit is that the CD80 expressed after inflammatory stimulus on podocytes can initiate the recombination of cellular actin of podocyte and result in the production of transient urine protein. Because of the Treg cells and the cytokines secreted by Treg cells (cytotoxic T-lymphocyte antigen-4, interleukin-10, *β*-transforming growth factor, and the regulation of podocyte per se), the sensitivity of the expressed CD80 can decline, and therefore the urine protein can be controlled. However, if the regulatory effect described above was disturbed, the sustained expression of CD80 could lead to sustained urine protein, which is the second hit [8].

Although the CD80 expressed by podocytes can lead to urine protein, Slavik et al. [11] found that the CD80 could be spotted from renal biopsy in the FSGS patients, but no elevation of urine CD80 was found. Therefore, the urine protein was not correlated with the severity of PNS. Instead, CD80 from the podocytes may exert a protective effect on podocytes, which could avoid detrimental effects. These findings were consistent with our study findings.

Our study found that the expression of urine CD80 in recurrent phase of SSNS patients was significantly higher than of those in remission as well as the control groups, while CD80 in the remission phase of SSNS was normal (7 cases of the patients investigated in our study can serve as before-after comparison, the result of which can further prove such point). This indicated that the elevation of CD80 was related to the recurrence of SSNS. It is known that most of the MCD types that were involved in children are SSNS, which supports the findings of our study.

FSGS is the commonest pathological change of end-stage renal disease, which is characterized by focal and segmental glomerular sclerosis, with or without formation and adhesion of capillary inner foam cells [23]. Due to its significant damage to the podocytes, urine proteins were likely to be detected. In patients with primary nephrotic syndrome, immunofluorescence showed that urine CD80 was primarily expressed on the surface of podocytes [17]. In contrast to this, because of the severe damage of podocytes, the expression of CD80 was declined, which leads to the urine CD80 that is less than that of MCD patients.

To further explore whether urinary CD80 levels are associated with the frequent relapse of nephrotic syndrome, our study analyzed the differences between the expressions of CD80 in frequent relapse of SSNS in relapse group and not frequent relapse of SSNS in relapse group. Results revealed that there was no significant difference observed between the groups. Similar comparison in remission phase was also insignificant, which indicated that the elevation of CD80 was not related to the frequency of recurrence.

Above all, our study found that urinary CD80 was related to the recurrence of SSNS, but not related to the frequency of recurrence. On the other hand, there is no correlation between urinary CD80 and 24-hour urinary protein. The elevation of urinary CD80 indicates the recurrence of SSNS, but it could not serve as a liable prognostic factor for predicting the frequent recurrence, and therefore more studies should be conducted to deduce the results.

## Figures and Tables

**Figure 1:**
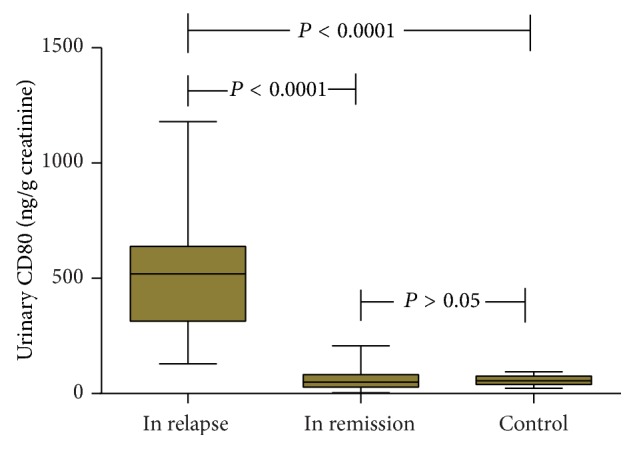
Comparison of urinary CD80 (ng/g creatinine) between and among SSNS patients in relapse and in remission and controls.

**Figure 2:**
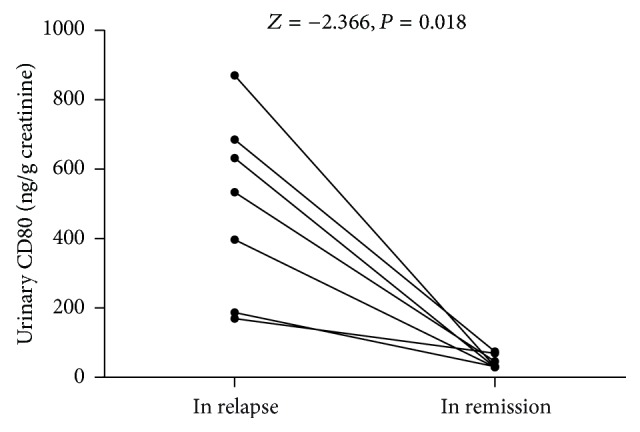
Comparison of urinary CD80 (ng/g creatinine) between the same patients in relapse and in remission.

**Figure 3:**
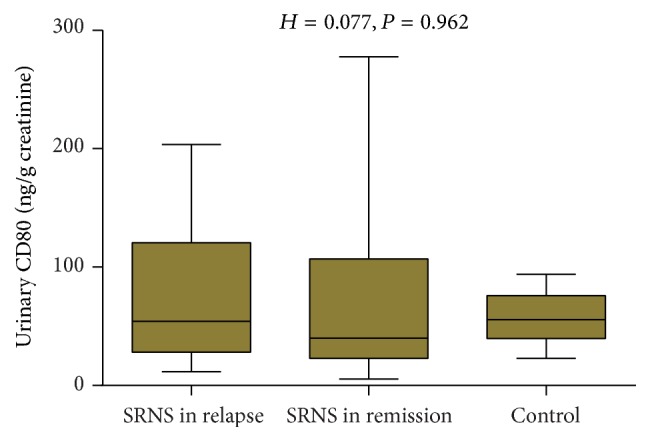
Comparison of urinary CD80 (ng/g creatinine) between and among SRNS patients in relapse and in remission and controls.

**Figure 4:**
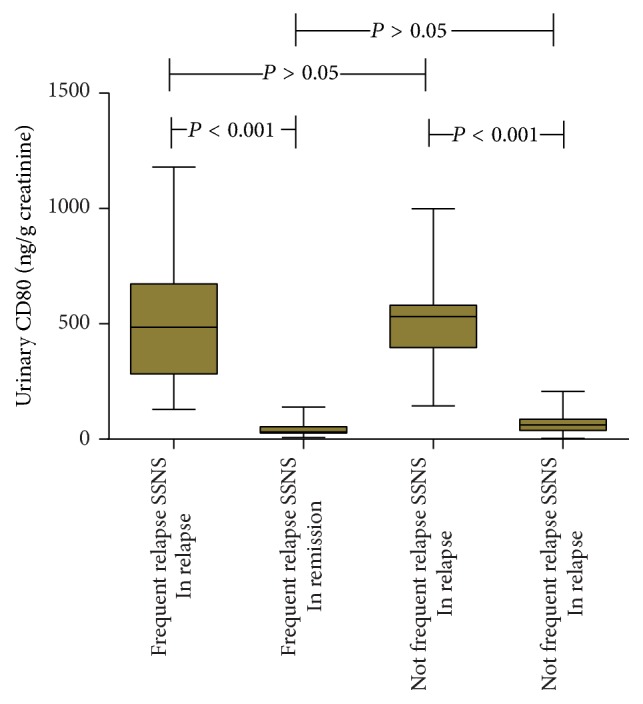
Comparison of urinary CD80 (ng/g creatinine) between and among patients in frequent relapse SSNS (in relapse and in remission) and not frequent relapse SSNS (in relapse and in remission).

**Figure 5:**
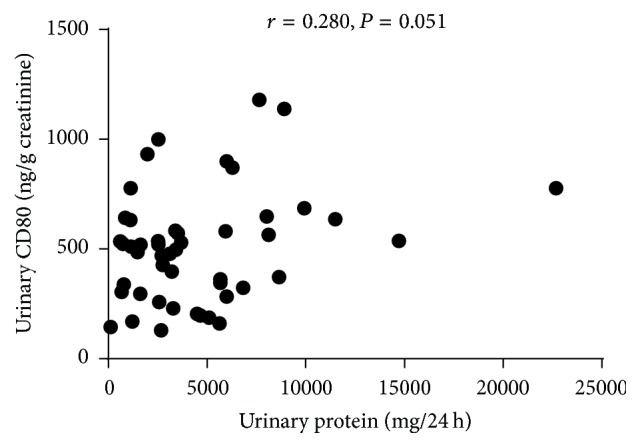
Correlation between urinary CD80 (ng/g creatinine) and 24-hour urinary protein (mg/kg).

**Table 1:** Comparison between and among SSNS in relapse group, SSNS in remission group, and the control group with basic information (*X* ± *S*).

Group	*n*	Age (year)	Males/females (case)	Serum albumin (g/l)	eGFR (ml/min/1.73 m2)
Control	25	5.7 ± 2.9	20/5	38.8 ± 3.8	180.3 ± 75.2
SSNS in relapse	49	6.7 ± 3.2	40/9	17.2 ± 6.4	170.4 ± 43.3
SSNS in remission	64	7.0 ± 3.5	52/12	39.9 ± 4.7	174.6 ± 35.3

*H* (*χ*2)		3.137	0.384		0.752
*P*		0.208	0.825		0.686

**Table 2:** Comparison between and among SRNS in relapse group, SRNS in remission group, and the control group with basic information (*X* ± *S*).

Group	*n*	Age (year)	Males/females (case)	Serum albumin (g/l)	eGFR (ml/min/1.73 m2)
SRNS in remission	8	6.2 ± 4.5	8/0	38.8 ± 3.8	164.1 ± 24.2
SRNS in relapse	7	7.4 ± 3.1	5/2	39.0 ± 6.1	157.4 ± 21.9
Control	25	5.7 ± 2.9	20/5	13.2 ± 5.9	180.3 ± 75.2

*H* (*χ*2)		1.717	2.571		0.301
*P*		0.424	0.276		0.860

**Table 3:** Comparison between and among patients in frequent relapse SSNS (in relapse and in remission) and not frequent relapse SSNS (in relapse and in remission) (*X* ± *S*).

Group	*n*	Age (year)	Males/females (case)	Serum albumin (g/l)	eGFR (ml/min/1.73 m^2^)
Frequent relapse SSNS					
In relapse	31	7.3 ± 3.1	25/6	17.3 ± 6.5	164.8 ± 43.2
In remission	26	8.4 ± 3.4^ab^	23/3^ab^	41.1 ± 4.1	175.2 ± 35.5^ab^
Not frequent relapse SSNS					
In relapse	18	5.7 ± 3.2^ab^	15/3^ab^	17.4 ± 6.4	180.9 ± 44.1^ab^
In remission	38	6.2 ± 3.2	29/9	39.1 ± 5.0	174.1 ± 35.6

^a^Compared with the frequent relapse SSNS in relapse group, *P* > 0.05. ^b^Compared with not frequent relapse SSNS in remission group, *P* > 0.05.
